# Development of a multimorbidity health conditions outcome index for caloric restriction interventional studies in older adults: a preliminary investigation in an observational cohort study

**DOI:** 10.1007/s11357-025-01708-4

**Published:** 2025-05-31

**Authors:** Michael E. Miller, Haiying Chen, Mark A. Espeland, Fang-Chi Hsu, Denise K. Houston, Anne B. Newman, W. Jack Rejeski, Barbara J. Nicklas, Stephen B. Kritchevsky

**Affiliations:** 1 Wake Forest University School of Medicine, Winston Salem, NC USA; 2 School of Public Health, University of Pittsburgh/UPMC, Pittsburgh, PA USA; 3 Department of Health and Exercise Sciences, Wake Forest University, Winston Salem, NC USA; 4 Division of Public Health Sciences, 525@Vine, 5th Floor, Medical Center Blvd., Winston-Salem, NC 27157 USA

**Keywords:** Older adults, Caloric restriction, Composite outcomes

## Abstract

Using multiple sources, we provide the conceptual justification and statistical support for a multimorbidity outcome associated with obesity-related conditions, which we term the Health Conditions Index (HCI). This index was designed to capture the health effects of multi-year studies of caloric restriction for older adults with BMIs in the overweight or obesity classification. We used a subset of participants in the Health, Aging and Body Composition Cohort Study to evaluate multiple aspects of the index and its components over 5 years of follow-up. The 937 participants in the subset had an average age of 73 years and a BMI of 30.7 kg/m^2^; 53% were female and 43% were Black; 80% were hypertensive and 14% had type 2 diabetes. Results demonstrated that the components of the index were consistently related to initial BMI and percent body fat on cohort entry, generally showed an increasing prevalence over the 5-year follow-up, and, as a composite index, exhibited an association between faster progression and higher initial levels of age, BMI, and percent body fat. Further, the initial HCI was associated with a statistically significant increase in the rate of mortality over 5 years of follow-up (hazard ratio = 1.22, 95% CI 1.04 to 1.44). By using outcomes like the HCI, clinical trials of caloric restriction in older adults may gain a better understanding of how intentional weight loss relates to future risk of multiple chronic conditions associated with aging.

**Supplementary information:**

The online version contains supplementary material available at 10.1007/s11357-025-01708-4.

## Introduction

Multimorbidity, the co-occurrence of multiple adverse health conditions, rises exponentially with age and, beginning at age 70, its incidence is approximately 10% per year [1]. Older adults with multimorbidity use a vastly disproportionate share of health care resources. In 2018, the 40% of Medicare beneficiaries with 4 or more conditions accounted for 78% of Medicare expenditures [2]. Obesity (BMI ≥ 30 kg/m^2^) is a major risk factor for serious age-related diseases like osteoarthritis, cardiovascular disease, and diabetes. The odds of multimorbidity are sevenfold times higher in persons with obesity than in those of normal weight, and even those in the overweight range (25 ≤ BMI < 30 kg/m^2^) have an increased risk of diseases [3, 4].

The prevalence of obesity among older adults (65 + years) in the USA has almost doubled over the last three decades, increasing from 22% in 1988–1994 to 40.2% in 2017–2018 [5]. Furthermore, over one-third (34.4%) of adults aged 75 and older have obesity. In addition, the number of older adults in the USA is rapidly increasing from ~ 35 million in 2000 to an estimated 73 million by 2030, comprising 20% of the USA population [5]. These projections suggest a concomitant rise in the number of older adults with obesity and multimorbidity. Therefore, interventions that reduce age-related multimorbidity either through the treatment of overweight/obesity or slowing the aging process are a high priority.

In older adults, 5 to 18 months of moderate behaviorally based caloric restriction leads to metabolic and functional improvements (e.g., lower IL-6, blood pressure, fasting glucose, better insulin sensitivity, and faster gait speed) [6–19]. However, the long-term prospect is unclear, with few relevant studies. The Action for Health in Diabetes (Look AHEAD) trial randomized 5145 adults aged 45–76 with overweight or obesity and type 2 diabetes to receive an intensive lifestyle intervention or an enhanced usual care condition (diabetes support and education) over 9–11 years. The lifestyle intervention arm targeted a 10% weight loss and increasing physical activity to ≥ 175 min/week. The lifestyle intervention was associated with the remission of diabetes and reduced rates of multimorbidity and frailty, better mobility function and physical health-related quality of life, and lower health care utilization [20–25]. The promise of the long-term benefit of weight loss on all-cause mortality is also suggested for middle- and older-aged persons undergoing bariatric surgery [26–30]. A meta-analysis suggests that bariatric surgery can increase life expectancy by 5.1 years in those without diabetes [31]. Whether this magnitude of benefit pertains to older persons is unclear.

In this report, we present the background, rationale, and results for a multimorbidity outcome, which we term the Health Conditions Index (HCI). The purpose of the index is to capture the benefits of caloric restriction (CR) over both the short and long term for use as an outcome in long-term human CR clinical trials. The HCI is composed of 29 conditions for which obesity or overweight increase risks. Without intervention, the prevalence of various components of the index is expected to increase with time in older individuals with overweight or obesity. We used the Health, Aging and Body Composition Cohort Study (Health ABC) to evaluate multiple aspects of the index and its components over 5 years of follow-up and estimate the expected change in the index in older adults who have an indication for obesity treatment.

## Methods

### Identification of weight-related multimorbidity conditions

The health conditions considered were drawn from expert consensus, clinical opinion, large data pooling projects, Mendelian randomization studies, and longer-duration clinical trials including CR. Examples of such reports on associated conditions are shown in Online Resource Table 1. Candidate components were reviewed individually and were selected based on the published literature and expert opinion applying five criteria (see Online Resource Table 2): (1) obesity/weight-relatedness; (2) age-relatedness; (3) reduction in the risk for, or severity of, the component through intentional weight loss; (4) association of the onset/severity of the component with aging-related biomarkers; and (5) occurrence typically leads to changes in clinical care or decreased quality of life. This review resulted in the selection of 29 health conditions that might be considered chronic diseases, of which nine are potentially reversible (see Table 1).

**Table 1:** Health Conditions Index component areas

Chronic diseases	Potentially reversible conditions
1. Acute coronary syndromes/CHD (including CHD death)	21. Hypertension (SPB > 130, or DPB > 80, or anti-hypertensive meds)
2. Incident deep vein thrombosis/embolism	22. Type 2 diabetes (HbA1c ≥ 6.5%; meds)
3. Stroke and stroke death (including revascularization for neurologic symptoms)	23. Obstructive sleep apnea (AHI > 15; or use of CPAP machine)**
4. Decompensated congestive heart failure	24. Knee/hip replacement or debilitating knee or hip pain (HOOS-12 or KOOS-12 < 40)
5. Chronic kidney disease (eGFR < 60, by Cystatin-C)	25. Depressive symptoms (CES-D > 16 or anti-depression meds)
6. Weight-related cancers* (*N* = 14)	26. Slowness (gait < 0.8 m/s)
20. Atrial fibrillation**	27. Lower limb weakness (5-chair stand > 15 s, no arms permitted)
	28. Exercise intolerance/major mobility disability (inability to walk 400 m)
	29. Tiredness/fatigue

*Cancers include esophagus, colon, rectum, kidney, pancreas, uterus, ovary, breast, stomach, liver, gall bladder, meningioma, thyroid, and multiple myeloma; each cancer is counted separately

**Items not available in the Health ABC dataset

### The Healthy Aging and Body Composition Cohort Study

The Health ABC study was a prospective cohort study of 3075 community-dwelling, high-functioning older adults who were 70–79 years of age at the time of enrollment [32]. Baseline study visits occurred between 1997 and 1998, after the protocol was approved by the Institutional Review Boards at both study sites. All participants provided written informed consent. Eligibility requirements included reporting no difficulty for (a) walking up 10 steps without resting, (b) walking one-quarter mile, or (c) performing activities of daily living. Additionally, participants were required to have no intention to move outside the area for 3 years and no known life-threatening cancer or end-stage chronic disease such as chronic obstructive pulmonary disease (COPD) requiring oxygen or renal failure requiring dialysis. Blood-based biomarkers measured at baseline included the following: fasting total plasma cholesterol (mg/dL), fasting plasma triglycerides (mg/dL), fasting plasma LDL (calculated, mg/dL), fasting plasma HDL (mg/dL), HbA1c, serum C-reactive protein (ug/mL), cystatin C (mg/L), IL6 (pg/mL), TNF-α I soluble receptor (pg/mL), TNF-α II soluble receptor (pg/mL), and leptin (ng/mL). This report uses baseline data and follow-up data on outcomes from five additional annual visits (years 1–5). Weight was measured without shoes or heavy clothing using a balance beam scale. Height was measured using a wall-mounted stadiometer. Body mass index was calculated as weight (kg)/height (m^2^). Percent body fat was measured using whole-body dual-energy absorptiometry (DXA).

We applied inclusion/exclusion criteria to the initial Health ABC sample to create a cohort of individuals who would be indicated for obesity treatment. We first included participants in our qualifying cohort if they either (a) had obesity (BMI of 30 to 37 kg/m^2^) or (b) were overweight (BMI 27 to 29.9 kg/m^2^) and at least one of the following morbidities:

- Elevated waist circumference (> 35″ in women, > 40″ in men)
- Hypertension 130–159 mmHg systolic or 80–99 mmHg diastolic (or on medication)
- Dyslipidemia (on medication or triglyceride > 200 mg/dl or total cholesterol > 240 mg/dl or LDL cholesterol > 160 mg/dl)
- Diabetes with control of HbA1c < 7.5%
- Other obesity-related morbidities, including prevalent coronary heart disease, definite ECG evidence of prior MI, and congestive heart failure, as well as cerebrovascular disease.

To match likely exclusion criteria for a future randomized trial, participants were excluded if they scored < 80 on the Modified Mini-Mental State Examination (3MS), had a Center for Epidemiological Studies-Depression scale (CESD) score ≥ 16, self-reported drinking more than 21 alcoholic drinks per week over the past year (< 1% of the full Health ABC sample), had uncontrolled hypertension (SBP > 160 or DBP > 100 and on medication for hypertension), used insulin, self-reported diabetes with HbA1c > 8.0%, or self-reported kidney disease, Parkinson’s disease, prior reported stomach surgery or hospitalization in the past year, or regular use of osteoporosis or diabetes medications. Application of the inclusion and exclusion criteria resulted in a cohort that we will subsequently refer to as the “qualifying cohort” in the remainder of this report, in contrast to those Health ABC participants that did not qualify (i.e., the “non-qualifying cohort”).

Within Health ABC, measures were identified to allow for the creation of indices capturing the presence/absence of HCI components at the baseline visit [33]. Atrial fibrillation and obstructive sleep apnea were not available in any year. Further, we attempted to score the overall HCI at each of the five subsequent annual visits. However, not all component areas were collected every year; thus, we decided to focus the analysis of HCI progression on data from the baseline, and years 3 and 5 when all components could contribute to the overall index. Once identified, chronic conditions were considered present at future visits. Events that occurred in continuous follow-up time (e.g., stroke, death) resulted in an increment to the index component indicator at the end of the yearly follow-up interval. Using this process, we were able to create the overall HCI at baseline and years 3 and 5 based on summing the 0/1 indicators for the presence of each condition.

Note that, due to reversible conditions (e.g., slowness, depressive symptoms), it is possible for an individual’s overall HCI value to decline over time. While the components of the HCI may vary by perceived severity and burden, and by the health care costs they may engender, we chose to weight them equally. For this application, we feel that each component captures an important feature of aging and that possible alternative weighting schemes are subjective relative to the construct to which they are anchored and may not generalize across settings and populations. The components are expected to have varying degrees of interrelatedness, in part not only due to the criteria we used for selecting them, but also due to relationships with an underlying construct of aging and aging-related processes.

### Health ABC index components

*Acute coronary syndromes/coronary heart disease (CHD)—including CHD death* was defined using an adjudicated classification of definite myocardial infarction (definite MI) or angina as the primary reason for hospitalization. A diagnosis of MI required one of the following: (a) cardiac pain or ischemic symptoms and abnormal cardiac enzymes with either an evolving ST-T pattern or an equivocal ECG pattern or (b) an evolving or diagnostic ECG pattern and abnormal cardiac enzymes. A diagnosis of angina required (a) a diagnosis of angina from a physician and treatment for angina, (b) symptoms of chest pain, pressure, or anginal equivalent treated with coronary artery bypass graft or percutaneous transluminal coronary angioplasty, (c) anginal symptoms with coronary angiography showing > 70% obstruction of any coronary artery, or (d) anginal symptoms with ST depression of more than 1 mm on exercise stress testing. CHD death required an underlying cause of death due to CHD (adjudicated). Baseline prevalent CHD was based on self-reported history of MI with the use of antianginal medications, self-reported history of angina with the use of antianginal medications, self-reported history of coronary revascularization procedures, or MI, angina, and revascularization procedures confirmed by Health Care Financing Administration (HCFA) codes within 5 years of the baseline exam.

*Incident deep vein thrombosis (DVT)/pulmonary embolism (PE)* was classified as incident if recorded as the primary reason for a hospitalization after the baseline examination. The history of DVT or PE prior to baseline was not assessed.

*Stroke and stroke death* was defined using an adjudicated classification of definite stroke as the primary reason for hospitalization and required all of the following: rapid onset of neurologic deficit attributed to obstruction or rupture of the arterial system, deficit lasting greater than 24 h (unless death intervenes), no evidence of cause due to tumor, trauma, infection, or other non-ischemic causes, new CT/MRI lesions consistent with clinical presentation. Stroke death required an underlying cause of death due to stroke. Baseline prevalent stroke was based on a self-reported history of stroke.

*Decompensated congestive heart failure (CHF)* was defined using an adjudicated classification of definite CHF as the primary reason for hospitalization based on (a) physician diagnosis of CHF and treatment including both a diuretic and digitalis or a vasodilator or (b) cardiomegaly and pulmonary edema on chest x-ray, evidence of a dilated ventricle and global or segmental wall-motion abnormalities with decreased systolic function either by echocardiography or contrast ventriculography. Baseline CHF was based on self-reported history of CHF.

*Chronic kidney disease (CKD)* was based on eGFR < 60 mL/min/1.73 m^2^ calculated *by Cystatin-C* (mg/L) with adjustment for age, sex, and race [34–36]:

$$eGFR=127.7\times{\textit{Cystatin}-C}^{-1.17}\times\textit{age}^{0.13}\times1.06\left(\textit{if black}\right)\times0.91\left(\textit{if female}\right)$$

eGFR was available at baseline and years 2, 3, and 5. Once a person was deemed to have CKD, it was considered present in future years.

*Weight-related cancers* included cancer diagnoses of the esophagus, colon, rectum, kidney, pancreas, uterus, ovary, breast, stomach, liver, gall bladder, thyroid, and multiple myeloma [37]. Because a participant may be enrolled with a history of one of the selected cancers but develop another type years later, each cancer was counted separately. Additionally, each of these cancers is thought to have differing mechanisms by which obesity increases the risk. Newly diagnosed incident cancer was based on an adjudicated primary cancer site confirmed by a pathology report, whereas baseline cancer was based on a self-reported history of cancer. Esophageal, pancreatic, and liver cancer, and multiple myeloma in the past 3 years at the time of screening were part of Health ABC exclusion criteria because of their poor prognosis.

Classification of *hypertension* used SPB ≥ 130 mmHg, DPB ≥ 80 mmHg, or self-reported hypertension with the use of anti-hypertensive medication at baseline and follow-up. Note that for year 3, medications reported in year 2 had to be used due to the absence of collected medications in year 3.

*Type 2 diabetes* relied on self-report of diabetes and current use of diabetes medication or fasting glucose ≥ 126 mg/dl, collected at baseline and in years 1, 3, and 5. Note that for year 3, medications reported in year 2 had to be used due to the absence of collected medications in year 3.

*Knee/hip replacement or debilitating knee pain* was defined as a history of knee or hip replacement, or a self-report of extreme knee pain in the past 30 days while walking on a flat surface, going up or down stairs, at night while in bed, standing, or getting in/out of a car/chair. Not all components of this measure were available in all years, and the presence of hip/knee replacement was defined as ongoing in future years, after the initial occurrence.

*Depressive symptoms* were defined based on the 10-item Center for Epidemiological Studies Depression (CESD-10) scale with a cut-point of ≥ 9 defining high symptomatology or currently taking medications for depression, as collected at baseline and in years 2–5. Note that for year 3, medications reported in year 2 had to be used due to the absence of collected medications in year 3.

*Slow gait speed* was obtained on a course of 4 or 6 m and defined as a gait speed < 0.8 m/s. The test consisted of walking at a usual pace, and two trials were performed; the fastest time was used in the analyses. For clinic visits in all years, the test was performed over 6 m, whereas a 4-m test was used for home visits.

*Lower body weakness* was based upon performance on a repeated chair stand test. Participants were asked to stand up and sit down five times from a chair as quickly as possible with their arms folded across their chest [38]. Inability to do 5 chair stands within 15 s or being unable to complete 5 chair stands was an indicator of weakness. This test was conducted at baseline and in years 3 and 5.

*Exercise intolerance/major mobility disability* was defined as the inability to walk 400 m. This inability was captured at baseline and in years 3 and 5, when the participant was unable to do the fast-paced 400-m walk due to the following: (1) meeting 400 m exclusion criteria at the visit, (2) inability to perform the initial 2-min walk, (3) 2-min walk completed, but 400 m not attempted, or (4) 400-m walk not completed.

*Tiredness* was defined using two items from the CES-D questionnaire. This condition required participant self-report on two of the 20 items from the CES-D questions, specifically that “I felt that everything I did was an effort” or “I could not get going” with a frequency of “Much” or “Most or all of the time” on a Likert scale, with total response options including “Rarely or None of the time,” “Some of the time,” “Much of the time,” and “Most or All of the time.” This information was available at baseline and in years 2, 3, and 5.

### Analytical methods

A flow diagram was used to describe how the Health ABC cohort was divided into those who met the inclusion and exclusion criteria (i.e., qualifying cohort) and to provide details on the yearly follow-up. Descriptive statistics were used to characterize the demographics, blood lipid levels, and physical function for participants by qualifying cohort membership. The phi coefficient for 2 × 2 tables was calculated as a measure of association between the HCI components. Spearman correlation coefficients were used to measure the association between baseline serum biomarkers and the overall HCI (and change in the index).

For the complete Health ABC cohort and separately for the qualifying cohort, logistic regression was used to explore the association that BMI and percent body fat had with each component of the index at baseline, using odds ratios (95% CI) to express the magnitude of the associations. For this analysis, the separate cancer diagnoses were collapsed into any cancer versus no cancer because few participants had > 1 cancer diagnosis. Because history of DVT or PE was not assessed at baseline, this variable was excluded from these analyses. These models were fit both with and without adjustment for sex as a potential confounder. Similarly, univariable and multivariable proportional hazards regression were used to explore the hazard ratio relating the overall baseline HCI score (and separate baseline index components) to death over 5 years. In the multivariable models, all available (i.e., DVT was excluded) index components were entered into the predictive model together.

To estimate the 5-year progression rate for the overall HCI, we focused on data from baseline and years 3 and 5 when all HCI components were available. The proportion of Health ABC participants with each index component was plotted against follow-up time to portray trends. For the overall HCI, we fit a mixed effects model with a random person effect and fixed effects for the inclusion/exclusion group, a continuous effect for time from baseline, and the interaction between the inclusion/exclusion group and the time variable. The overall progression rates within the qualifying and non-qualifying cohorts were also estimated from mixed effects models. In addition, within the qualifying cohort, we fit models that explored interactions between follow-up time and baseline age (< 75, 75 +), baseline BMI (< 30, 30 +), and baseline percent body fat (used as a continuous variable and adjusted for sex in the model) effects, allowing estimation of progression rates within subgroups for those meeting the inclusion/exclusion criteria.

Outcome data were missing for both individual binary index components and for whole visits (e.g., due to participant drop-out). At baseline, due to items being collected at an in-person visit, seven index components had some missing data (with the maximum level occurring for history of CHD, with 1.8% of participants having missing information). At baseline, we assumed that components that were missing were absent. At follow-up visits, we took two approaches to incorporating missing items. In what we would consider a conservative approach relative to estimating progression through time, we assumed that a missing component was equal to the absence of the diagnosis/trait and calculated the overall HCI score. This was done only for participants that were missing < 50% of the index components. The overall HCI was set to missing for participants with more than 50% of items missing at a specific visit. As an alternative approach, we used SAS PROC MI to implement a fully conditional specification (FCS) imputation method for binary data with arbitrary missing patterns [39, 40] to impute the missing binary HCI indicators. This process included all indicators across baseline and years 3 and 5 and generated 100 multiply imputed datasets. Estimates of parameters and standard errors were then combined across multiple imputations to produce estimates. We relied on our mixed effect models, which are valid under an assumption of missing are random [41], to account for visits where the overall HCI was missing, in contrast to the missed HCI items.

## Results

Online Resource Fig. 1 provides the flow diagram defining the cohort that was used for analysis and those that did not qualify for the analytical cohort. Health ABC initially enrolled 3075 participants between the ages of 70 and 79. Nine hundred and thirty-seven participants (30.5%) met the inclusion and exclusion criteria and were included in the qualifying cohort. Of these 937, a total of 91 participants died by the 5-year anniversary of their baseline visit. Missed participant contacts (other than death) ranged from 1% at the first annual visit to 2.9% after 5 years of follow-up; some proxy contacts were obtained in each year.

Participants in the qualifying cohort had an average age of 73.3 years and 42.6% self-identified as Black (see Table 2). Approximately 53% were women; the average BMI was 30.7 kg/m^2^ and the average percent body fat obtained by DXA was 38.5%. As expected, those that did not qualify had lower BMI and percent body fat, smaller waist circumference, lower triglycerides and LDL, and higher HDL and leptin. There was little difference in walking speed between the groups.

**Table 2:** Demographics of Health ABC participants by qualifying cohort

	*Did not qualify (n* = *2138)*	*Qualified (n* = *937)*	*P-value*
Age, yrs (mean (SD))	73.76 (2.90)	73.34 (2.80)	< 0.01
Race/ethnicity (*N* (%))			0.49
White	1256 (58.7)	538 (57.4)	
Black	882 (41.3)	399 (42.6)	
Sex (*N* (%))			0.37
Men	1048 (49.0)	443 (47.3)	
Women	1090 (51.0)	494 (52.7)	
BMI (kg/m^2^) (mean (SD))	25.95 (4.78)	30.67 (2.93)	< 0.01
BMI groups (*N* (%)			< 0.01
< 27 kg/m^2^	1569 (73.4)	0 (0.0)	
27 to < 30 kg/m^2^	253 (11.8)	469 (50.1)	
30 to < 40 kg/m^2^	267 (12.5)	468 (49.9)	
≥ 40 kg/m^2^	49 (2.3)	0 (0.0)	
Percent body fat (mean (SD))	33.48 (7.67)	38.45 (7.03)	< 0.01
Waist circumference (in) (mean (SD))	37.95 (4.92)	42.04 (4.66)	< 0.01
Lab measures (mean (SD))			
Total cholesterol (mg/dL)	201.54 (38.02)	205.84 (39.96)	< 0.01
Triglycerides (mg/dL)	133.00 (81.73)	151.01 (83.78)	< 0.01
LDL (calculated, mg/dL)	120.10 (34.43)	125.38 (35.20)	< 0.01
HDL (mg/dL)	55.37 (17.50)	50.82 (15.28)	< 0.01
HbA1c	6.41 (1.26)	6.26 (0.74)	< 0.01
C-reactive protein (ug/mL)	2.95 (4.99)	3.13 (4.10)	0.35
Cystatin C (mg/L)	1.04 (0.38)	1.05 (0.25)	0.66
IL6 (pg/mL)	2.79 (2.60)	2.91 (2.62)	0.27
TNF-a I soluble receptor (pg/mL)*	1590.3 (576.35)	1581.6 (512.00)	0.79
TNF-a II soluble receptor (pg/mL)*	3563.8 (1373.0)	3608.4 (2227.4)	0.64
Leptin (ng/mL)	12.63 (12.46)	19.87 (14.15)	< 0.01
Physical function measures (mean (SD)			
6-m or 4-m walk (m/s)	1.18 (0.24)	1.17 (0.22)	0.71
Overall Health Conditions Index (HCI)	2.09 (1.43)	1.94 (1.27)	< 0.01
HCI components			
Acute coronary syndromes (*N* (%)	396 (18.5)	161 (17.2)	0.37
Stroke and stroke death (*N* (%)	55 (2.6)	17 (1.8)	0.20
Decompensated CHF (*N* (%)	69 (3.2)	26 (2.8)	0.50
Weight-related cancers (*N* (%)			0.28
0	2002 (93.6)	887 (94.7)	
1	131 (6.1)	46 (4.9)	
2	5 (0.2)	4 (0.4)	
Hypertension (*N* (%)	1697 (79.4)	747 (79.7)	0.83
Type 2 Diabetes (*N* (%)	397 (18.6)	130 (13.9)	< 0.01
Knee/hip replacement or hip pain (*N* (%)	134 (6.3)	79 (8.4)	0.03
Depressive symptoms (*N* (%)	249 (11.6)	51 (5.4)	< 0.01
Slow gait (*N* (%)	102 (4.8)	26 (2.8)	0.01
Weakness (chair stands) (*N* (%)	64 (3.0)	17 (1.8)	0.06
Exercise intolerance (*N* (%)	530 (24.8)	221 (23.6)	0.47
Tiredness (*N* (%)	182 (8.5)	52 (5.5)	< 0.01

*Available on ~ 50% of participants

The proportion of participants with each individual component score and the mean overall HCI score is provided in Table 2. Participants in the qualifying cohort had a mean index value of 1.94 (SD = 1.27) compared to 2.09 (SD = 1.43) for those not qualifying. Among qualifiers, ~ 80% had hypertension, ~ 24% had exercise intolerance, ~ 17% had a history of an acute coronary syndrome, and ~ 14% had type 2 diabetes. There were several notable differences between the groups, with lower percentages in the qualifying cohort primarily related to exclusion criteria (e.g., less depression and type 2 diabetes).

Online Resource Fig. 2 presents the distribution of the HCI at baseline by qualifying group, illustrating a slight shift towards higher scores in the distribution of those meeting the qualifying criteria. Pairwise phi coefficients to measure the associations between HCI components at baseline were modest, with a range of − 0.03 to 0.32 for the 78 coefficients that were calculated within the whole Health ABC sample (highest two values were as follows: depression and tiredness = 0.32; slowness and weakness = 0.20). When limited to participants in the qualifying cohort, the range of phi was reduced to − 0.06 to 0.16, which indicates that the index components measure relatively unrelated events/limitations.

### Associations between BMI and percent body fat with HCI at baseline

**Fig. 1:**
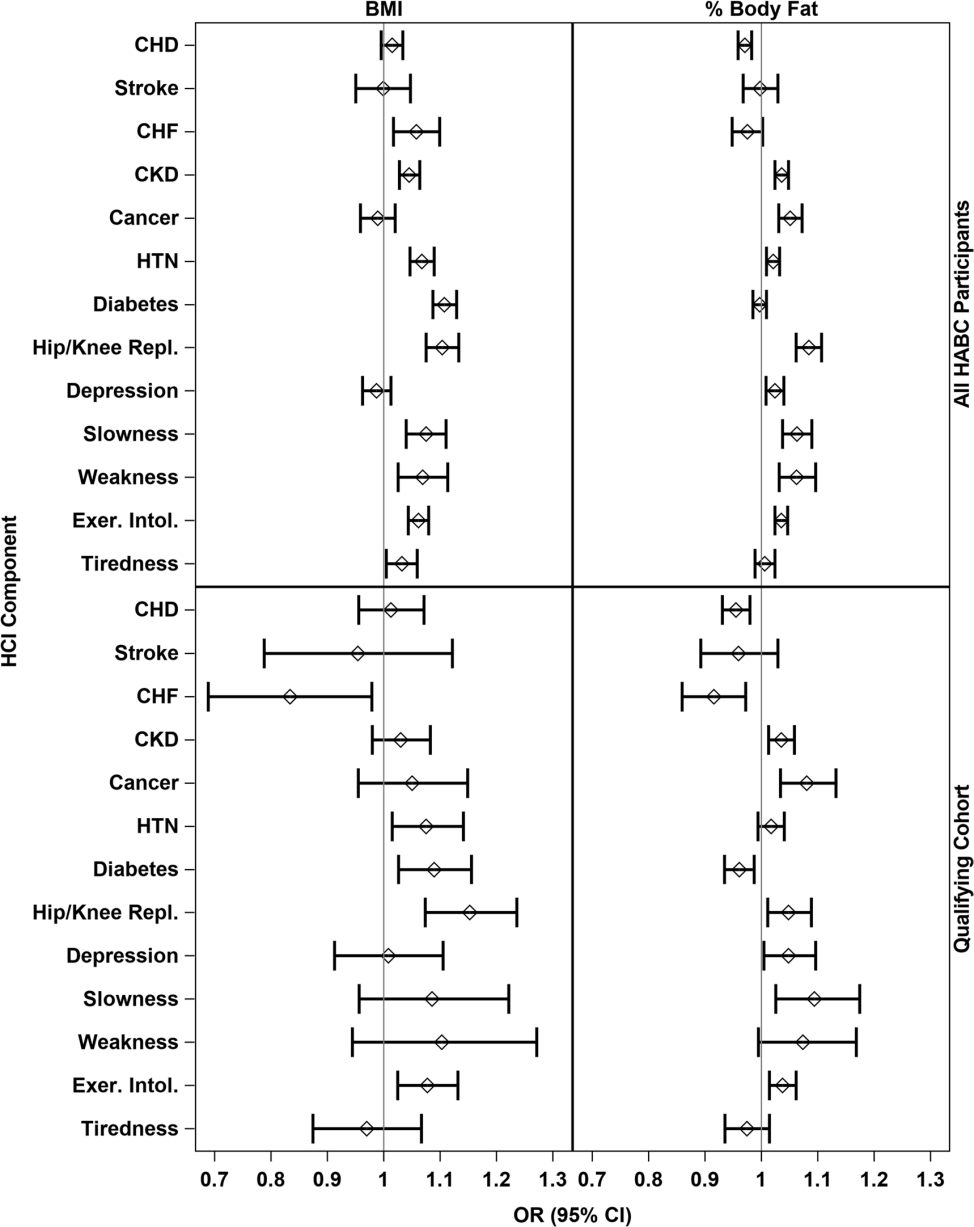
Odds ratios (95% CIs) for a one unit difference in BMI and percent body fat for each HCI component at baseline of the Health ABC study

At baseline, the Spearman correlations between the HCI and BMI and percent body fat were 0.13 (*p* < 0.001) and 0.08 (p-0.02), respectively, within the qualifying cohort, and 0.20 (*p* < 0.001) and 0.23 (*p* < 0.001) within the whole Health ABC study cohort. Results from logistic regression analyses using both BMI and percent body fat to predict each HCI component are presented in Fig. 1. In this figure, we present odds ratios (95% CIs) for a one-unit difference in BMI and percent body fat separately for each HCI indicator. Analyses are presented for the whole Health ABC cohort and for the qualifying cohort. For BMI and the full Health ABC cohort (top left panel), we observed ORs ≥ 1 for all HCI components except cancer and depressive symptoms. Within the qualifying cohort (bottom left panel), stroke, CHF, and tiredness had estimated ORs < 1; adjustment for sex had a minimal impact on these relationships with BMI (see Online Resource Fig. 3). For percent body fat and the complete Health ABC cohort (top right panel), all estimated ORs are approximately 1 or greater, except for CHD and CHF; when adjusted for sex (see Online Resource Fig. 3), these two ORs were attenuated to ~ 1 or became greater than 1. A similar pattern was observed within the qualifying cohort (bottom right panel) except that CHD, stroke, CHF, diabetes, and tiredness had estimated ORs < 1 when not adjusted for sex, but CHD and diabetes became attenuated when adjusted for sex.

### Baseline association of HCI with 5-year mortality

**Fig. 2:**
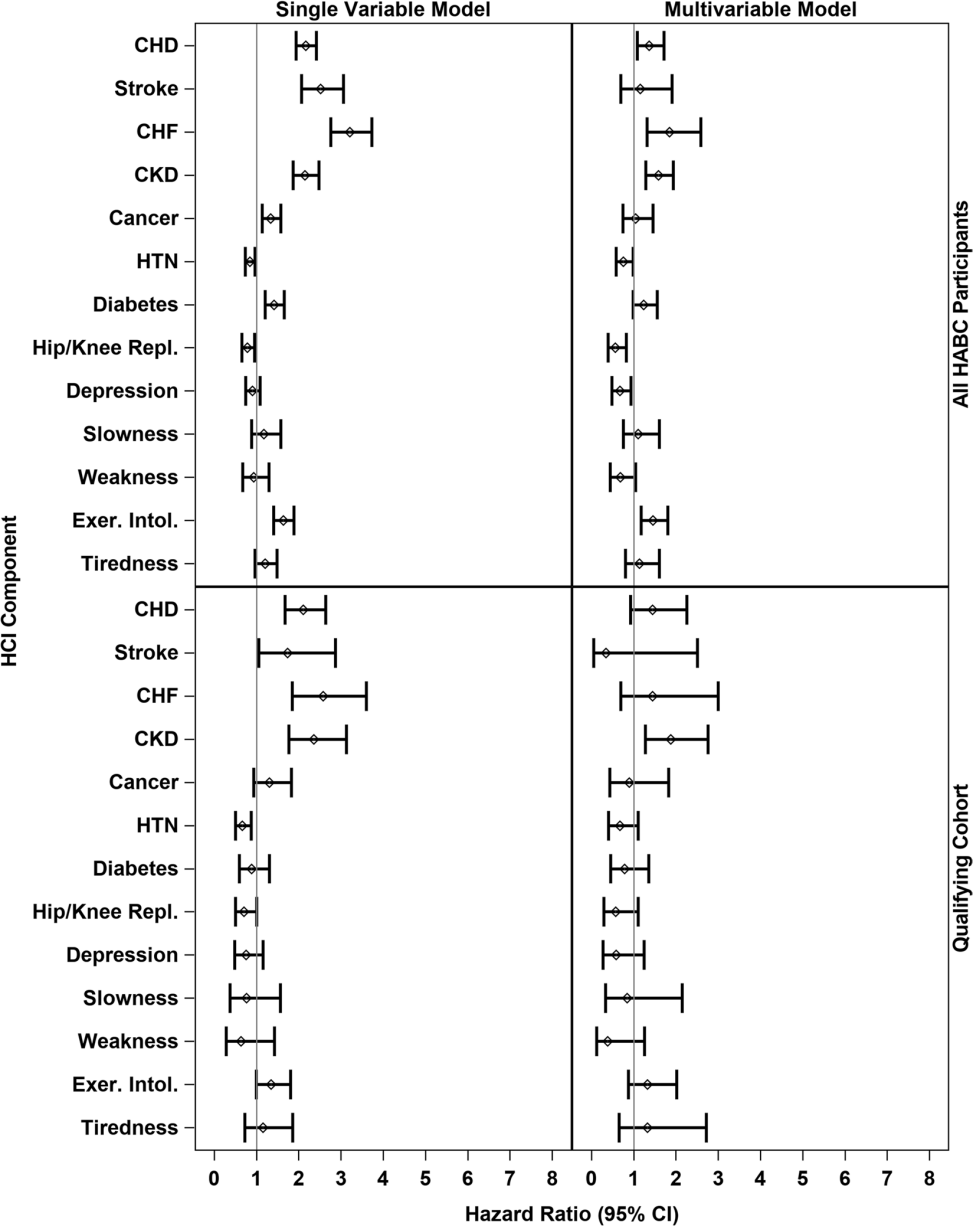
Hazard ratios for association of each baseline HCI component to 5-year mortality

As a predictor of the time until death, the baseline HCI score had a hazard ratio of 1.32 (95% CI 1.24 to 1.40) for a one-unit difference within the complete Health ABC cohort and 1.22 (95% CI 1.04 to 1.44) within the qualifying cohort. Further, Fig. 2 presents the results from proportional hazards regression relating each baseline HCI component to death over 5 years of follow-up. Results from models including only a single predictor are presented in the first column panel, and multivariable results, including all baseline HCI components, are presented in the second column panel. In the single predictor models, for all Health ABC participants, all HCI components had estimated hazard ratios (HR) ≥ 1 except for the hip/knee replacement/pain indicator. Most relationships were attenuated when all components were added, with CHD, CHF, and CKD remaining nominally significant predictors (*p* < 0.05). Similar trends were seen in the qualifying cohort, with wider confidence intervals on estimated HRs. We note that among components that were used in the inclusion/exclusion criteria to create the qualifying cohort, the HRs for diabetes, knee/hip replacement/pain, and depressive symptoms are the most attenuated.

### Progression of HCI over 5 years of follow-up

**Fig. 3:**
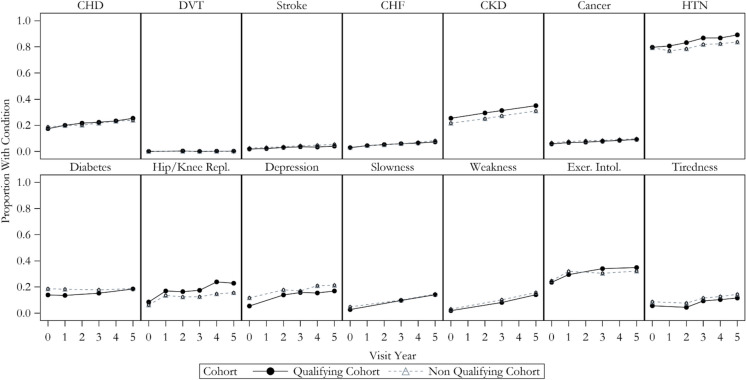
Proportion of participants with each HCI component over time

The 5-year overall proportion of participants with each index component of the HCI remained stable or increased with time (Fig. 3). In the qualifying cohort, approximately 55% added at least one point, and 30% had at least a two-point increase in their HCI (see Online Resource Fig. 4). In the non-qualifying participants, these two values were 50% and 25%, respectively.

Using the “conservative” HCI score in which a missing index component was equal to the absence of the diagnosis/trait, the estimated rate of progression from the mixed model analysis was 0.193 units/year (95% CI 0.175, 0.211) in the qualifying cohort and 0.150 units/year (95% CI 0.138, 0.162) in the non-qualifying cohort (*p* < 0.001 for a test of equality between these progression rates). When multiple imputation was applied to account for item nonresponse prior to computing a person’s HCI and subsequently running mixed effects models on the multiply imputed datasets, the progression rate was estimated to be 0.222 units/year (95% CI 0.203, 0.242) in those in the qualifying cohort and 0.189 units/year (95% CI 0.177, 0.202) in the non-qualifying cohort.

The estimated progression rate was 0.197 units/year (95% CI 0.172, 0.221) in those aged < 75 at cohort entry and 0.271 units/year (95% CI 0.237, 0.306) in the 75 + group (*p* < 0.001 for a test of interaction) in the qualifying cohort, based on multiple imputation. Likewise, those with higher BMI or percent body fat also tended to have higher rates within the qualifying group. For example, within the qualifying cohort, those with BMI < 30 kg/m^2^ had an estimated progression rate of 0.187 units/year (95% CI 0.160, 0.215), whereas those entering with a BMI of ≥30 + kg/m^2^ had a rate of 0.257 units/year (95% CI 0.229, 0.285; *p* < 0.001 for a test of interaction). Additionally, those entering the cohort with less than 40% body fat had an estimated progression rate of 0.206 units/year (95% CI 0.179, 0.234), whereas those entering with 40% or greater body fat had an annual rate of 0.241 units/year (95% CI 0.212, 0.270; *p* = 0.09 for test of interaction).

### Association with initial levels of inflammatory/aging biomarkers

A cross-sectional analysis found that all 6 inflammatory biomarkers were positively associated with the HCI value at baseline (See Online Resource Table 3; Spearman correlations ranged from 0.11 for leptin to 0.39 for cystatin C, *p* < 0.001 for all). Baseline levels of the biomarkers were not associated with a 5-year change in the HCI.

## Discussion

We provided the background and rationale for the use of a multimorbidity Health Conditions Index (HCI) as a novel outcome for testing interventions (such as caloric restriction) in clinical trials of older adults with overweight and obesity. Our analyses demonstrated that within a subgroup of the Health ABC cohort of older adults that would be targeted for caloric restriction or obesity treatment, the components of the index were consistently related to initial BMI and percent body fat on cohort entry, generally showed an increasing prevalence over 5 years of follow-up, and as a composite score showed an association between faster progression and higher initial levels of age, BMI, and percent body fat. Importantly, the baseline HCI was associated with a statistically significant increase in the rate of mortality over 5 years of follow-up. When considering these results in the context of prior evidence that CR in humans may be associated with increases in longevity, the HCI could constitute an important geroscience-themed outcome for studies involving CR or obesity treatment in older adults [42, 43].

The HCI shares some similarities to frailty indices (FI) based on accumulated deficits, but it also has some important differences. Frailty indices are used to measure changes in age-related health status and aging over time. The components used in FI vary, but they should usually include elements that are age-related, health relevant, not ubiquitous in the population, and cover a range of systems [44, 45]. The HCI also includes a variety of components, which are chosen based on evidence that they are adversely related to both age and obesity. Because of the interest in CR as an approach to influence aging biology to improve health, the HCI adds the additional criteria that the components be related to aging-relevant biomarkers. We observed that the HCI correlates with the age-related biomarkers IL-6, CRP, cystatin-C, and TNF-alpha soluble receptors 1 and 2. A major difference between frailty indices and the HCI is that the HCI is designed to capture the benefits of CR in clinical trials, while the FI is not focused on CR or weight loss.

The literature on the use of composite indices in trials emphasizes challenges to interpretation when all elements of the composite do not respond in a similar way to a given intervention [46]. An index that includes elements that move in opposite directions for a given intervention also undermines statistical power. Our data illustrate that HCI components in the Health ABC study are related to obesity status at baseline. Furthermore, in addition to other criteria, evidence that CR or weight loss influences the risk of, or severity of, the element was also applied, although for some of the elements (e.g., rarer cancer types), these data are not available. With these differences stated, we are aware of several clinical trials that used a frailty index as an exploratory outcome [47]. The Look AHEAD trial compared an intensive lifestyle intervention including caloric restriction and increased exercise to an education and diabetes support program in persons with overweight or obesity and type 2 diabetes and found a significant reduction in the frailty index among those in the intensive lifestyle intervention arm [48]. However, randomized placebo-controlled trials of aspirin, vitamin D, omega-3 fatty acids, and canakinumab— a monoclonal antibody that inhibits IL-1beta— showed no effect on frailty indices [49–51].

The HCI has many advantages compared to other potential single component trial outcomes: (1) it captures the wide range of benefits consistent with animal, observational, and clinical trial data; (2) each element has direct relevance to public health, clinical decision making, or quality of life and thus is more compelling than fluid biomarker-based outcomes; (3) it is congruent with obesity as a major determinant of multimorbidity; (4) half of its elements can improve with weight loss, providing a wider dynamic range relative to composites based on only incident or worsening chronic disease; (5) it can require a smaller sample size than an endpoint focused on a single disease or class of diseases; and (6) it provides the basis for linking changes in aging/obesity biomarkers to the onset of additional age-related health conditions, helping to validate these biomarkers as potential surrogates in the context of a CR intervention [52]. In addition to these advantages, a majority of the HCI components can be collected through means of electronic health records, self-report, or limited home visits, serving as a potentially cost-efficient means of capturing important information in participants that may experience intermittent bouts of disability. A limitation is that it has been designed to capture benefits and must be assessed separately against the potential harms associated with CR and intentional weight loss in older adults [53].

### Limitations

The Health ABC dataset is a cohort study, and as such, we cannot infer causality of the observed relationships. In addition, while the Spearman correlation between baseline BMI and HCI was 0.13 in the qualifying cohort, limiting the range of BMI by applying the proposed entry criteria for a randomized clinical trial clearly resulted in attenuation of the association compared to the overall Health ABC cohort, where this correlation was 0.20. Other unmeasured factors may also differ between those that enroll in a cohort study and those that are randomized in a clinical trial. Importantly, it is the progression rates of HCI associated with different baseline and future levels of BMI (or percent body fat) resulting from a CR program that are of primary importance for understanding potential effect sizes in a future study. Weight loss in older adults enrolled in observational studies like Health ABC is often associated with illness and potentially with increases in HCI over time. In fact, among 2708 Health ABC participants in which intent for weight loss was indicated at baseline, 27% reported they were trying to lose weight; however, no significant difference in actual weight loss was found after 1 year of follow-up between participants intending and not intending to lose weight [54]. Additionally, dietary intake was only assessed at 1-year follow-up in Health ABC, and, thus, we were unable to assess change in dietary intake between baseline and 1-year follow-up. While the index as a whole has not been shown to be responsive to weight loss, items were selected because of published data indicating responsiveness to intentional weight loss.

The index includes items related to diseases and states of functional decline including slow gait speed, the inability to complete a 400-m walk, failure on a chair stand task, and symptoms/feelings suggestive of chronic tiredness (fatigue and depressive symptoms). Readers may wonder why we did not consider outcomes that could potentially capture improvements in self-esteem, confidence, or overall quality of life. They were not included in the index, not because they are not important to older adults, but because they are not age-related. The purpose of the index is to capture conditions that are both age- and weight-related to provide insight into whether CR might slow the progression of age-related diseases and health conditions. Further, some studies of older adults have shown that weight loss in the absence of increased physical activity does not affect objective indices of physical function nor lead to significant changes in quality of life [8, 55]. In comprehensive obesity treatment, which includes increased physical activity in conjunction with weight loss, changes in health-related quality of life occur, but only for the dimension of physical functioning, not mental health [56]. We would note that any trial of CR in older adults would likely include additional potential benefits as secondary outcomes.

Missing outcomes resulting from missing index components and/or missed visits may complicate analyses and constitute a limitation involved with using composite outcomes. We illustrated how a multiple imputation approach may address missed index components and have used the properties of mixed effects models, which account for missingness dependent on previously collected outcomes or covariates, to handle missed visits [41]. Additionally, as previously mentioned, composite indices of this nature are not without limitations when considered within the framework of randomized trials [46, 57]. Rules guiding interim testing of the composite HCI outcome may not be straightforward because some components may also require monitoring from a safety perspective (e.g., cardiovascular mortality). One approach might be to conduct interim testing only on mortality (or another pre-specified safety outcome), while reserving final analyses for the composite [58].

Consistent with analytical considerations for time-to-event outcomes, competing risks can play a role in the interpretation and analysis of an index outcome. Competing risks consist of situations where a participant may experience failure from one cause, but that failure precludes observation of other events. For example, a participant that experiences CHD death cannot subsequently have a stroke. When survival analysis is being used on a time-until-event outcome, multiple approaches have been recommended to reduce bias in the estimation of disease risk [59, 60]. Likewise, for an index-based HCI outcome, biased estimation of the progression rates can occur in situations where mortality from one cause precludes observation of other events. Additionally, accounting for death in a primary analysis may also pose challenges due to the missing HCI outcomes after death not being missing at random. Joint modelling of longitudinal and time-to-event data has been proposed for use when analyzing longitudinal responses truncated by death and can provide a viable approach to addressing this problem [61–66]. More recently, a class of shared parameter models to jointly model a normally distributed outcome over time and multiple causes of failure using cumulative incidence functions has been proposed [67]. We anticipate that joint models of this type may be useful to account for competing risks in analyses of outcomes like the proposed HCI.

Similarly, but not necessarily fitting into the competing risk paradigm, is a situation where someone that is unable to walk 4 m clearly cannot do a performance-based test of their ability to walk 400 m. Adjudication of events that have a cascading effect on other outcomes (e.g., inability to walk 4 m infers inability to walk 400 m) resulting in disease clusters should be part of the process of creating such an index, with rules set up to increment outcomes that rely on the ability to perform other outcomes (e.g., the exercise intolerance/major mobility disability indicator automatically received an increment when failure to walk 4 m occurs).

### Future directions

Many index-based outcomes have considered an approach that emphasizes different components through using weights to give greater emphasis to components that substantially impact underlying constructs like quality of life or mortality risk. Approaches to developing weights for index components may include statistical approaches such as principal components or simulation, approaches that assign weights relative to each component’s relationship to some underlying construct, or expert opinion [68–70]. It has been pointed out in the area of social research that methods of weighting and aggregation of index components are often the subject of criticism [71]. We have chosen to not weight components due to the somewhat subjective nature of weighting, which is an approach that is similar to what others have published in constructing frailty indices [44]. Furthermore, if some rare items are given greater weights, it might become necessary to tailor recruitment criteria to focus recruitment on participants expected to have those types of events to keep the study sample size manageable. This enrichment may reduce the generalizability of both the sample and the results. Further research to evaluate weighting approaches for an index like the HCI may be best carried out by evaluating the perceived importance of various outcomes, as determined by focus groups of older adults that would be eligible for a caloric restriction intervention.

Ideally, a study of the effects of CR in older adults needs to use an outcome that will lead to an understanding of how intentional weight loss relates to future risk of multiple chronic conditions, both through the direct benefits of weight loss and the indirect benefits that may relate to effects on underlying aging processes. There is no standard list of conditions to choose from for CR trials of older populations; thus, extensive background literature reviews and exploration of population-based databases are extremely important. Prior research has emphasized that the choice of the number and type of health conditions can greatly influence reported multimorbidity prevalence in certain groups of people [72]. Clarity in reporting and understanding the disease clusters around obesity in older adults are two critical steps in developing a more systematic approach to effective interventions for those affected [73, 74]. The HCI was not designed as a general index to be used with any intervention affecting underlying aging processes but was customized specifically for CR trials. We sought to develop a geroscience-relevant outcome that could show both reduced mortality from age-related causes and reduced progression of age-related health conditions and be relevant and meaningful to older adults with obesity or who are overweight and have at least one comorbid condition. However, the approach we took may provide a way to develop outcomes suited to other geroscience interventions (e.g., rapalogs) in other populations.

## Supplementary information

Below is the link to the electronic supplementary material.

ESM 1 (DOCX 606 KB)

## Data Availability

The data for this study can be requested from the Health ABC study public use dataset (https://healthabc.nia.nih.gov).
