# Supplementary material for: Development of a multimorbidity health conditions outcome index for caloric restriction interventional studies in older adults: a preliminary investigation in an observational cohort study
Source: GeroScience. 2025 May 31;48(1):235–52. doi: 10.1007/s11357-025-01708-4 (PMC12972406; doi:10.1007/s11357-025-01708-4)
Supplement: Supplementary file 1 — (DOCX 606 KB) [file 11357_2025_1708_MOESM1_ESM.docx]

Online Resource For:

**Development of a Multimorbidity Health Conditions Outcome Index for Caloric Restriction Interventional Studies in Older Adults: A Preliminary Investigation in an Observational Cohort Study**

Michael E. Miller^1^; Haiying Chen^1^; Mark A. Espeland^1^; Fang-Chi Hsu^1^; Denise K. Houston^1^; Anne B. Newman^2^; W. Jack. Rejeski^3^; Barbara A. Nicklas^1^; Stephen B. Kritchevsky^1^

1. Wake Forest University School of Medicine, Winston Salem, NC, USA
2. School of Public Health, University of Pittsburgh/UPMC, Pittsburgh, PA, USA
3. Department of Health and Exercise Sciences, Wake Forest University, Winston Salem, NC, USA

**Corresponding author:**

Michael E. Miller

Division of Public Health Sciences

525@Vine, 5th Floor

Medical Center Blvd.

Winston-Salem, NC 27157

[mmiller@wakehealth.edu](mailto:mmiller@wakehealth.edu)

ORCID: 0000-0002-3903-6655

| **Online Resource Table 1. Identification of Weight Related Health Conditions** | | |
| --- | --- | --- |
| **Source** | **Population** | **Outcome** |
| 2013 AHA / ACC / TOS [1] | All Adults | Type 2 Diabetes, Hypertriglyceridemia / Elevated LDL-C, Hypertension prevention and treatment |
| Health Effects of Overweight and Obesity in 195 Countries over 25 years[2] | All Adults | Cancers: (Esophageal; Colorectal, Gallbladder and Biliary tract, Pancreatic, Breast, Uterine, Ovarian, Kidney, Thyroid, Leukemia), Ischemic Heart Disease, Ischemic Stroke, Hemorrhagic Stroke, Hypertensive Heart Disease, Diabetes, Osteoarthritis (knee / hip), Low Back Pain |
| UK Biobank Mendelian Randomization Studies[3, 4] | Men / Women 40-69 | Mortality: Cardiovascular Disease, Stomach, and Esophageal Cancer;  Health Conditions: Aortic Valve Stenosis, Heart Failure, Deep Vein Thrombosis, Arterial Hypertension, Peripheral Arterial Disease, Coronary Artery Disease, Atrial Fibrillation, Pulmonary Embolism; Cancers (Endometrial, Squamous Cell Lung; Ovarian; Liver; Stomach) |
| **Clinical Trials** |  |  |
| TONE[5] | 585 men & women aged 60-80 with obesity, mild or normotensive and on 1 bp med | Diagnosis of high blood pressure, treatment with antihypertensives or a CVD event; Weight loss (5 kg by 6 months) associated a 30% reduction in the outcome |
| Look AHEAD[6–8] | Men and women 45-76 with type 2 diabetes | 34% reduction in depressive symptoms, 2.92 mmHg Lower SBP, 31% reduction in very high-risk kidney disease, 16% reduction in rate of obesity-related cancers (ns); no effect on CVD; better self-reported function at 4 years & faster gait speed at 8 years. |

| **Online Resource Table 2. Summary of Diseases / Health Conditions Considered for Inclusion in the HCI** | | | | |
| --- | --- | --- | --- | --- |
| **Condition** | **BMI Related** | **Biomarker Related** | **Weight Loss Benefit** | **Age-Related** |
| Chronic Kidney Disease | Yes[9] | Yes[10] | Yes[9, 11] | Yes |
| Type 2 Diabetes | Yes | Yes[12] | Yes[13, 14] | Yes |
| Cancer | Yes; esophagus, colon, rectum, kidney, pancreas, uterus, ovary, post-menopausal breast, stomach, cardia, liver, gallbladder, meningioma, thyroid, and multiple myeloma[15] | Yes[16, 17] | Probable for ‘weight-related cancers’[8, 18] | Yes |
| Congestive Heart Failure (both pEF and rEF) | Yes[19]; pEF > rEF | Yes[20, 21] | Yes for exercise tolerance[22]; Semaglutide[23] | Yes |
| Acute Coronary Syndromes | Yes[24] | Yes[21] | Gastrectomy Reduces Risk[25];  Semaglutide[23] | Yes |
| Atrial Fibrillation | Yes[26] | Yes[27] | Yes[28] | Yes |
| Stroke (Ischemic & Hemorrhagic) | Yes[29] | Yes[30] | Bariatric Surgery[31]; Semaglutide[23] | Yes |
| Hypertension | Yes[32] | No[33] | Yes[5] | Yes |
| Dyslipidemia (Elevated LDL) | Yes[32] | Yes[34] | Yes[35] | No[36] |
| Peripheral Arterial Disease | No[37] | Yes[37] | For progression[38] | Yes |
| Sleep Apnea | Yes[39] | Yes[40] | Reduces Severity[39] | Yes[39] |
| Lung Disease (not COPD) | Mixed[41] | Yes[42, 43] | Mixed[44–46] | Yes[47] |
| COPD | Mixed[48] | Yes[49, 50] | Bariatric Surgery[51] | Yes[52] |
| VTE / PE | Yes[53, 54] | Unclear[55, 56] | Yes[57] | Yes[53, 54] |
| Anemia | Through iron metabolism[58, 59] | Yes[60] | Unclear[61, 62] | Yes[63] |
| Parkinson’s Disease | Maybe waist circumference[64, 65] | Yes, in those with a dx, prediction unclear[66, 67] | No relevant study identified | Yes[68] |
| Cognitive Impairment | Yes, but Age-Paradox[69] | Yes[70] | Mixed[71, 72] | Yes |
| Depression | Yes[73] | Yes[74] | Yes[75] | Bi-modal[76] |
| Lower extremity joint disease | Yes[77] | Yes[78] | Yes[79] | Yes |
| Hip Fracture | Inversely WHR maybe related[80, 81] | Yes[82, 83] | No sign. difference in Look AHEAD[84] | Yes |
| Weakness | Inversely[85] | Yes[86] | No short-term change | Yes |
| Slowness | Yes[87] | Yes[86] | Yes[88] | Yes |
| Low Energy | Yes[89, 90] | Yes[91, 92] | For Fatigue[93] | Yes |
| Sit-to-Stand | Yes[94] | Yes[86] | Yes[95] | Yes |
| Exercise Intolerance (ability to walk 400m) | Yes[96] | Severe limitation by self-report [97] | Benefits for Gait speed and self-reported mobility[98] | Yes |

| **Online Resource Table 3. Associations Between Inflammatory Biomarkers**  **and HCI** | | | |
| --- | --- | --- | --- |
|  | **Spearman’s Correlation Coefficient** | | |
| **Biomarker** | **Baseline HCI** | **Year 3 HCI – Baseline HCI** | **Year 5 HCI – Baseline HCI** |
| Baseline CRP | 0.16*** | <-0.01 | 0.06 |
| Baseline Cystatin C | 0.39*** | 0.01 | <-0.01 |
| Baseline IL6 | 0.25*** | 0.05 | 0.02 |
| Baseline TNF-a I | 0.33*** | 0.01 | -0.01 |
| Baseline TNF-a II | 0.29*** | 0.04 | 0.01 |
| Baseline Leptin | 0.11*** | 0.01 | 0.02 |

* p<0.05

** p<0.01

*** p<0.001

Online Resource Figure 1. Flow Diagram for Defining Health ABC Cohorts


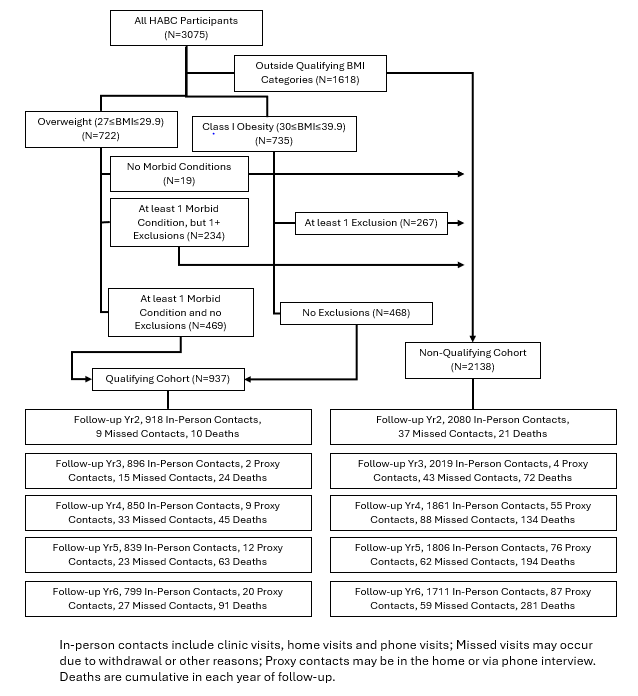


**Online Resource Figure 2. Distribution of HCI in Baseline**


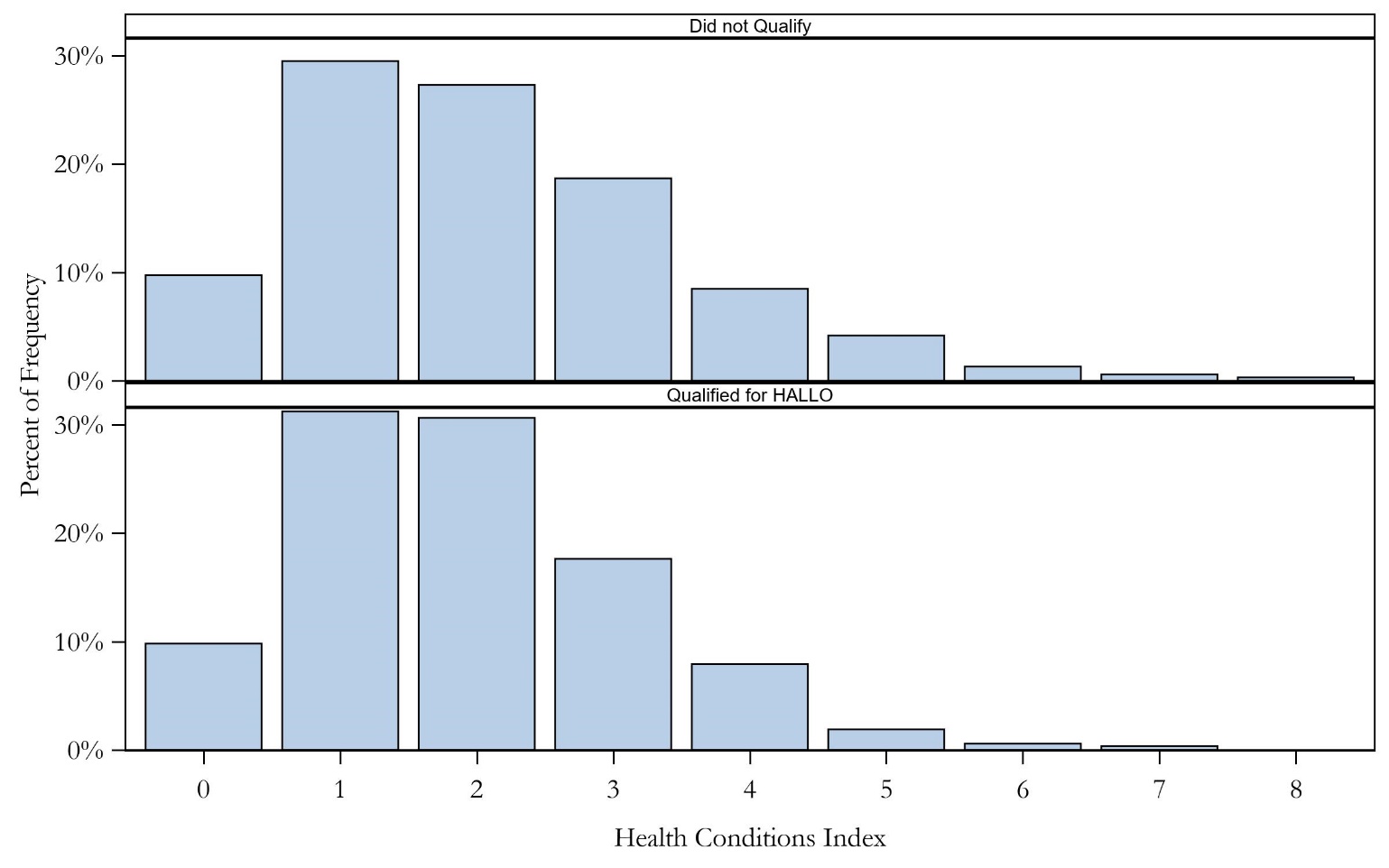


**Online Resource Figure 3. Association of BMI and % Body Fat to each HCI Component at Baseline**

**Adjusted for Sex**


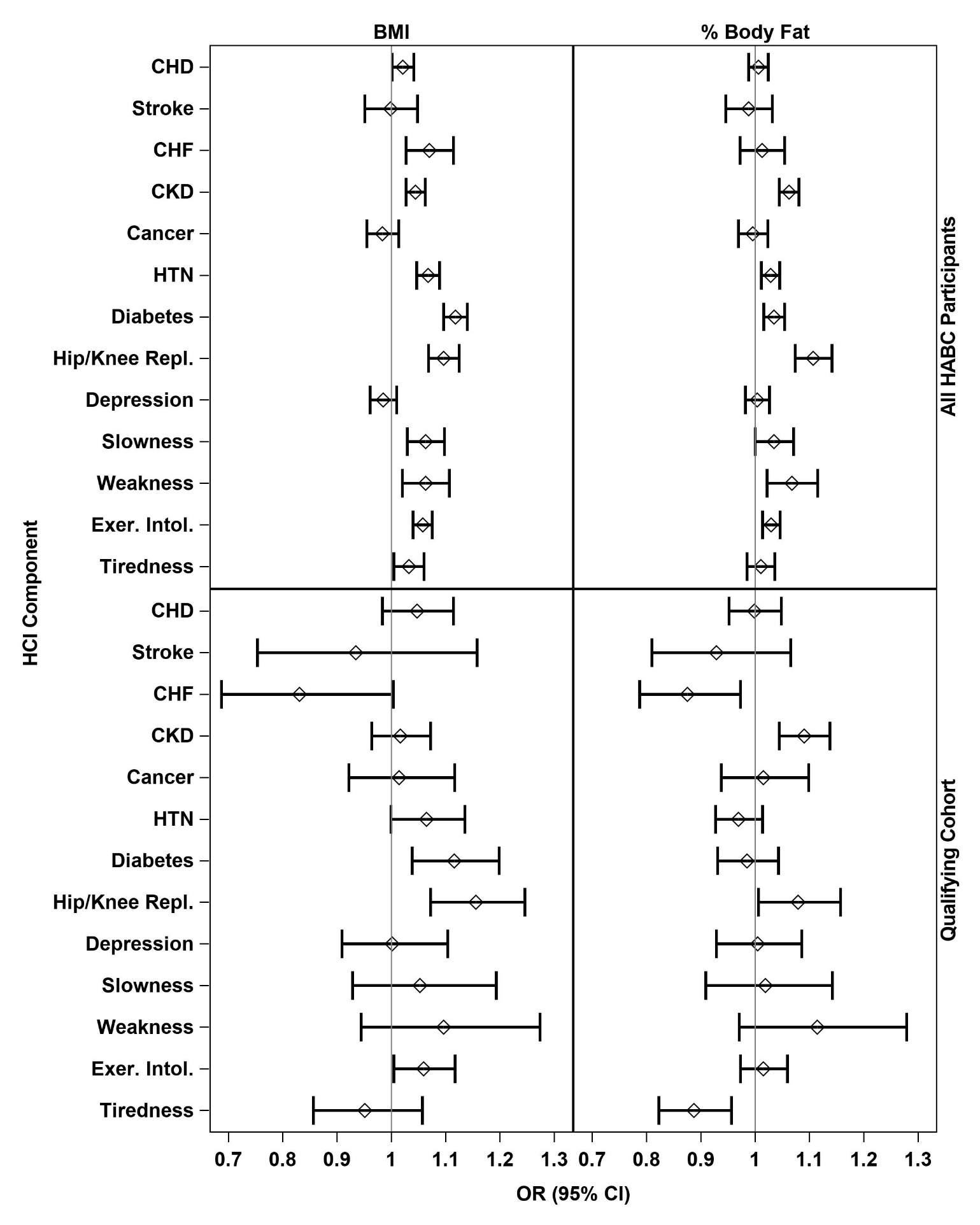


**Online Resource Figure 4. Distribution of 5-year Change in HCI**


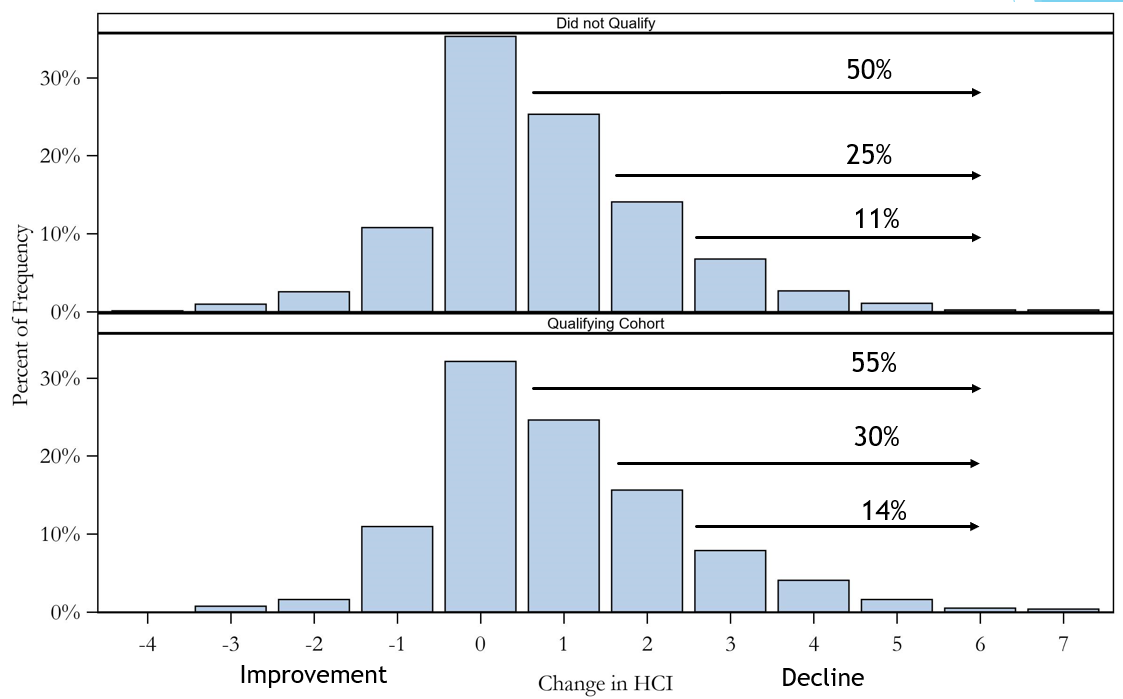


**Online Resource References**

1. Jensen MD (2013) AHA/ACC/TOS guideline for the management of overweight and obesity in adults: a report of the American College of Cardiology/American Heart Association Task Force on Practice Guidelines and The Obesity Society. Circulation 24;129(25 Suppl 2):S102-38:. https://doi.org/10.1161/01.cir.0000437739.71477.ee.

2. GBD 2015 Obesity Collaborators (2017) Health Effects of Overweight and Obesity in 195 Countries over 25 Years. N Engl J Med 6;377(1):13-27:. https://doi.org/10.1056/NEJMoa1614362.

3. Vithayathil M, Carter P, Kar S, et al (2021) Body size and composition and risk of site-specific cancers in the UK Biobank and large international consortia: A mendelian randomisation study. PLoS Med 29;18(7):e1003706: https://doi.org/10.1371/journal.pmed.1003706.

4. Larsson SC, Bäck M, Rees JMB, et al (2020) Body mass index and body composition in relation to 14 cardiovascular conditions in UK Biobank: a Mendelian randomization study. Eur Heart J 7;41(2):221-226:. https://doi.org/10.1093/eurheartj/ehz388.

5. Whelton PK, Appel LJ, Espeland MA, et al (1998) Sodium Reduction and Weight Loss in the Treatment of Hypertension in Older Persons: A Randomized Controlled Trial of Nonpharmacologic Interventions in the Elderly (TONE). JAMA 279:839. https://doi.org/10.1001/jama.279.11.839

6. Look A.H.E.A.D.Research Group (2013) Cardiovascular effects of intensive lifestyle intervention in type 2 diabetes. N Engl J Med 11;369(2):145-54:. https://doi.org/10.1056/NEJMoa1212914.

7. Rubin RR (2014) Impact of intensive lifestyle intervention on depression and health-related quality of life in type 2 diabetes: the Look AHEAD Trial. Diabetes Care Jun;37(6):1544-53: https://doi.org/10.2337/dc13-1928.

8. Look A.H.E.A.D.Research Group (2020) Intensive Weight Loss Intervention and Cancer Risk in Adults with Type 2 Diabetes: Analysis of the Look AHEAD Randomized Clinical Trial. Obesity Sep;28(9):1678-1686: https://doi.org/10.1002/oby.22936.

9. Jiang Z, Wang Y, Zhao X, et al (2022) Obesity and Chronic Kidney Disease. Am J Physiol Endocrinol Metab. https://doi.org/10.1152/ajpendo.00179.2022.

10. Salimi S, Shardell MD, Seliger SL, et al (2018) Inflammation and Trajectory of Renal Function in Community-Dwelling Older Adults. J Am Geriatr Soc Apr;66(4):804-811: https://doi.org/10.1111/jgs.15268.

11. Shaver LN, Beavers DP, Kiel J, et al (2019) Effect of Intentional Weight Loss on Mortality Biomarkers in Older Adults With Obesity. J Gerontol A Biol Sci Med Sci 12;74(8):1303-1309:. https://doi.org/10.1093/gerona/gly192.

12. Wang X, Bao W, Liu J, et al (2013) Inflammatory markers and risk of type 2 diabetes: a systematic review and meta-analysis. Diabetes Care Jan;36(1):166-75. doi:10 2337 12-0702. https://doi.org/10.2337/dc12-0702.

13. Diabetes Prevention Program Research Group (2002) Reduction in the Incidence of Type 2 Diabetes with Lifestyle Intervention or Metformin. N Engl J Med 346:393–403. https://doi.org/10.1056/NEJMoa012512

14. Tuomilehto J, Lindstrom J, Eriksson JG (2001) Prevention of type 2 diabetes mellitus by changes in lifestyle among subjects with impaired glucose tolerance. N Engl J Med 344:1343–1350. https://doi.org/10.1056/NEJM20010503344180

15. Lauby-Secretan B, Scoccianti C, Loomis D, et al (2016) International Agency for Research on Cancer Handbook Working Group. Body Fatness and Cancer–Viewpoint of the IARC Working Group. N Engl J Med 25;375(8):794–8:. https://doi.org/10.1056/NEJMsr1606602.

16. Rahimi GRM, Yousefabadi HA, Niyazi A, et al (2022) Effects of Lifestyle Intervention on Inflammatory Markers and Waist Circumference in Overweight/Obese Adults With Metabolic Syndrome: A Systematic Review and Meta-Analysis of Randomized Controlled Trials. Biol Res Nurs Jan;24(1):94-105: https://doi.org/10.1177/10998004211044754.

17. Lemmelä S, Wigmore EM, Benner C, et al (2022) Integrated analyses of growth differentiation factor-15 concentration and cardiometabolic diseases in humans. Elife 2;11:e76272: https://doi.org/10.7554/eLife.76272.

18. Aminian A, Wilson R, Al-Kurd A, et al (2022) Association of Bariatric Surgery With Cancer Risk and Mortality in Adults With Obesity. JAMA 28;327(24):2423-2433:. https://doi.org/10.1001/jama.2022.9009.

19. Ho JE, Enserro D, Brouwers FP, et al (2016) Predicting Heart Failure With Preserved and Reduced Ejection Fraction: The International Collaboration on Heart Failure Subtypes. Circ Heart Fail. https://doi.org/10.1161/CIRCHEARTFAILURE.115.003116.

20. Libby P (2021) Inflammation during the life cycle of the atherosclerotic plaque. Cardiovasc Res 22;117(13):2525-2536:. https://doi.org/10.1093/cvr/cvab303.

21. Cesari M, Penninx BWJH, Newman AB, et al (2003) Inflammatory Markers and Onset of Cardiovascular Events: Results From the Health ABC Study. Circulation 108:2317–2322. https://doi.org/10.1161/01.CIR.0000097109.90783.FC

22. Kitzman DW, Brubaker P, Morgan T, et al (2016) Effect of Caloric Restriction or Aerobic Exercise Training on Peak Oxygen Consumption and Quality of Life in Obese Older Patients With Heart Failure With Preserved Ejection Fraction: A Randomized Clinical Trial. JAMA 5;315(1):36-46:. https://doi.org/10.1001/jama.2015.17346.

23. Lincoff AM, Brown-Frandsen K, Colhoun HM, et al (2023) Semaglutide and Cardiovascular Outcomes in Obesity without Diabetes. N Engl J Med 14;389(24):2221-2232:. https://doi.org/10.1056/NEJMoa2307563.

24. Arbab-Zadeh A, Nakano M, Virmani R, Fuster V (2012) Acute coronary events. Circulation 6;125(9):1147-56:. https://doi.org/10.1161/CIRCULATIONAHA.111.047431.

25. Benotti PN, Wood GC, Carey DJ, et al (2017) Gastric Bypass Surgery Produces a Durable Reduction in Cardiovascular Disease Risk Factors and Reduces the Long-Term Risks of Congestive Heart Failure. J Am Heart Assoc 23;6(5):e005126: https://doi.org/10.1161/JAHA.116.005126.

26. Wang TJ, Parise H, Levy D, et al (2004) Obesity and the risk of new-onset atrial fibrillation. JAMA 24;292(20):2471–7:. https://doi.org/10.1001/jama.292.20.2471.

27. Patel P, Dokainish H, Tsai P, Lakkis N (2010) Update on the association of inflammation and atrial fibrillation. J Cardiovasc Electrophysiol Sep;21(9):1064-70: https://doi.org/10.1111/j.1540-8167.2010.01774.x.

28. Pathak RK, Middeldorp ME, Meredith M, et al (2015) Long-Term Effect of Goal-Directed Weight Management in an Atrial Fibrillation Cohort: A Long-Term Follow-Up Study (LEGACY. J Am Coll Cardiol 26;65(20):2159-69:. https://doi.org/10.1016/j.jacc.2015.03.002.

29. Kernan WN, Inzucchi SE, Sawan C, et al (2013) Obesity: a stubbornly obvious target for stroke prevention. Stroke Jan;44(1):278-86: https://doi.org/10.1161/STROKEAHA.111.639922.

30. Georgakis MK, Malik R, Gill D, et al (2020) Interleukin-6 Signaling Effects on Ischemic Stroke and Other Cardiovascular Outcomes: A Mendelian Randomization Study. Circ Genom Precis Med Jun;13(3):e002872: https://doi.org/10.1161/CIRCGEN.119.002872.

31. Williams MD, Kim S, Sarran M, et al (2021) The effect of bariatric surgery on ischemic stroke risk. Surg Obes Relat Dis. Dec;17(12):1949-1955

32. Kotsis V, Jordan J, Micic D, et al (2018) Obesity and cardiovascular risk: a call for action from the European Society of Hypertension Working Group of Obesity, Diabetes and the High-risk Patient and European Association for the Study of Obesity: part A: mechanisms of obesity induced hypertension, diabetes and dyslipidemia and practice guidelines for treatment. J Hypertens Jul;36(7):1427-1440: https://doi.org/10.1097/HJH.0000000000001730.

33. Gordon JH, LaMonte MJ, Zhao J, et al (2021) The association between serum inflammatory biomarkers and incident hypertension among postmenopausal women in the Buffalo OsteoPerio Study. J Hum Hypertens Sep;35(9):791-799: https://doi.org/10.1038/s41371-020-00422-2.

34. Hong N, Lin Y, Ye Z, et al (2022) The relationship between dyslipidemia and inflammation among adults in east coast China: A cross-sectional study. Front Immunol 13:. https://doi.org/10.3389/fimmu.2022.937201.

35. Kraus WE, Bhapkar M, Huffman KM, et al (2019) 2 years of calorie restriction and cardiometabolic risk (CALERIE): exploratory outcomes of a multicentre, phase 2, randomised controlled trial. Lancet Diabetes Endocrinol Sep;7(9):673-683: https://doi.org/10.1016/S2213-8587(19)30151-2.

36. Downer B, Estus S, Katsumata Y, Fardo DW (2014) Longitudinal trajectories of cholesterol from midlife through late life according to apolipoprotein E allele status. Int J Environ Res Public Health 16;11(10):10663-93:. https://doi.org/10.3390/ijerph111010663.

37. Aday AW, Matsushita K (2021) Epidemiology of Peripheral Artery Disease and Polyvascular Disease. Circ Res 11;128(12):1818-1832:. https://doi.org/10.1161/CIRCRESAHA.121.318535.

38. Polonsky TS, Tian L, Zhang D, et al (2019) Associations of Weight Change With Changes in Calf Muscle Characteristics and Functional Decline in Peripheral Artery Disease. J Am Heart Assoc 2;8(13):e010890: https://doi.org/10.1161/JAHA.118.010890.

39. Chang JL, Goldberg AN, Alt JA (2022) International consensus statement on obstructive sleep apnea. Int Forum Allergy Rhinol. https://doi.org/10.1002/alr.23079.

40. Locke BW, Lee JJ, Sundar KM OSA and Chronic Respiratory Disease: Mechanisms and Epidemiology. Int J Environ Res Public Health 2022 Apr 30;19(9):5473: https://doi.org/10.3390/ijerph19095473.

41. Mafort TT, Rufino R, Costa CH (2016) Obesity: systemic and pulmonary complications, biochemical abnormalities, and impairment of lung function. Multidiscip Respir Med 11:28. https://doi.org/10.1186/s40248-016-0066-z

42. Oldham JM (2021) Interstitial Lung Abnormalities and Aging Biomarkers: A Mediation. Am J Respir Crit Care Med. 1;203(9):1058-1060

43. Sanders JL, Putman RK, Dupuis J, et al (2021) The Association of Aging Biomarkers, Interstitial Lung Abnormalities, and Mortality. Am J Respir Crit Care Med 1;203(9):1149-1157:. https://doi.org/10.1164/rccm.202007-2993OC.

44. Pugashetti J, Graham J, Boctor N, et al (2018) Weight loss as a predictor of mortality in patients with interstitial lung disease. Eur Respir J 16;52(3):1801289: https://doi.org/10.1183/13993003.01289-2018.

45. Comes A, Wong AW, Fisher JH, et al (2022) Association of BMI and Change in Weight With Mortality in Patients With Fibrotic Interstitial Lung Disease. Chest May;161(5):1320-1329: https://doi.org/10.1016/j.chest.2021.11.008.

46. Sekine A, Wasamoto S, Hagiwara E, et al (2021) Beneficial impact of weight loss on respiratory function in interstitial lung disease patients with obesity. Respiratory Investigation 59:247–251. https://doi.org/10.1016/j.resinv.2020.10.002

47. Rojas M, Mora AL, Kapetanaki M, et al (2015) Clinical Impact and Cellular and Molecular Pathways. Ann Am Thorac Soc Dec;12(12):S222-7: https://doi.org/10.1513/AnnalsATS.201508-484PL.

48. Fuller-Thomson E, Howden KEN, Fuller-Thomson LR, Agbeyaka S (2018) A Strong Graded Relationship between Level of Obesity and COPD: Findings from a National Population-Based Study of Lifelong Nonsmokers. J Obes. https://doi.org/10.1155/2018/6149263.

49. Su B, Liu T, Fan H, et al (2016) Inflammatory Markers and the Risk of Chronic Obstructive Pulmonary Disease: A Systematic Review and Meta-Analysis. PLoS One 22;11(4):e0150586: https://doi.org/10.1371/journal.pone.0150586.

50. Mercado N, Ito K, Barnes PJ (2015) Accelerated ageing of the lung in COPD: new concepts. Thorax May;70(5):482-9: https://doi.org/10.1136/thoraxjnl-2014-206084.

51. Cogollo VJ, Valera RJ, Botero-Fonnegra C, et al (2022) Bariatric surgery decreases hospitalization rates of patients with obstructive lung diseases: a nationwide analysis. Surg Obes Relat Dis Aug;18(8):1042-1048: https://doi.org/10.1016/j.soard.2022.04.013.

52. MacNee W (2016) Is Chronic Obstructive Pulmonary Disease an Accelerated Aging Disease? Annals ATS 13:S429–S437. https://doi.org/10.1513/AnnalsATS.201602-124AW

53. Gregson J, Kaptoge S, Bolton T, et al (2019) Emerging Risk Factors Collaboration. Cardiovascular Risk Factors Associated With Venous Thromboembolism. Emerging Risk Factors Collaboration Cardiovascular Risk Factors Associated With Venous Thromboembolism JAMA Cardiol 1;4(2):163-173:. https://doi.org/10.1001/jamacardio.2018.4537

54. Mi Y, Yan S, Lu Y, et al (2016) Venous thromboembolism has the same risk factors as atherosclerosis: A PRISMA-compliant systemic review and meta-analysis. Medicine (Baltimore Aug;95(32):e4495: https://doi.org/10.1097/MD.0000000000004495.

55. Setiawan B, Budianto W, Sukarnowati TW, et al (2022) Correlation of Inflammation and Coagulation Markers with the Incidence of Deep Vein Thrombosis in Cancer Patients with High Risk of Thrombosis. Int J Gen Med. https://doi.org/10.2147/IJGM.S372038.

56. Wang G, Wu BF, Zhao WJ, et al (2024) C-reactive protein is a predictor for lower-extremity deep venous thrombosis in patients with primary intracerebral hemorrhage. Eur J Med Res 6;29(1):311: https://doi.org/10.1186/s40001-024-01842-3.

57. Harrington LB, Benz L, Haneuse S, et al (2024) Bariatric Surgery and the Long-Term Risk of Venous Thromboembolism: A Population-Based Cohort Study. Obes Surg Jun;34(6):2017-2025: https://doi.org/10.1007/s11695-024-07236-y.

58. Dao MC, Meydani SN (2013) Iron biology, immunology, aging, and obesity: four fields connected by the small peptide hormone hepcidin. Adv Nutr 6;4(6):602-17:. https://doi.org/10.3945/an.113.004424.

59. Zhao L, Zhang X, Shen Y, et al (2015) Obesity and iron deficiency: a quantitative meta-analysis. Obes Rev Dec;16(12):1081-93: https://doi.org/10.1111/obr.12323.

60. Ferrucci L, Semba RD, Guralnik JM, et al (2010) Proinflammatory state, hepcidin, and anemia in older persons. Blood 6;115(18):3810-6:. https://doi.org/10.1182/blood-2009-02-201087.

61. Teng IC, Tseng SH, Aulia B, et al (2020) Can diet-induced weight loss improve iron homoeostasis in patients with obesity: A systematic review and meta-analysis. Obes Rev Dec;21(12):e13080: https://doi.org/10.1111/obr.13080.

62. Tussing-Humphreys LM, Nemeth E, Fantuzzi G, et al (2010) Decreased serum hepcidin and improved functional iron status 6 months after restrictive bariatric surgery. Obesity Oct;18(10):2010-6: https://doi.org/10.1038/oby.2009.490.

63. Gaskell H, Derry S, Andrew Moore R, McQuay HJ (2008) Prevalence of anaemia in older persons: systematic review. BMC Geriatr 8:. https://doi.org/10.1186/1471-2318-8-1.

64. Chen H, Zhang SM, Schwarzschild MA, et al (2004) Obesity and the risk of Parkinson’s disease. Am J Epidemiol 15;159(6):547-55:. https://doi.org/10.1093/aje/kwh059.

65. Chen J, Guan Z, Wang L, et al (2014) Meta-analysis: overweight, obesity, and Parkinson’s disease. Int J Endocrinol 2014:. https://doi.org/10.1155/2014/203930.

66. D’Ascenzo N, Antonecchia E, Angiolillo A, et al (2022) Metabolomics of blood reveals age-dependent pathways in Parkinson’s Disease. Cell Biosci 6;12(1):102: https://doi.org/10.1186/s13578-022-00831-5.

67. Paul KC, Binder AM, Horvath S, et al (2021) Accelerated hematopoietic mitotic aging measured by DNA methylation, blood cell lineage, and Parkinson’s disease. BMC Genomics 26;22(1):696: https://doi.org/10.1186/s12864-021-08009-y.

68. Hirsch L, Jette N, Frolkis A, et al (2016) The Incidence of Parkinson’s Disease: A Systematic Review and Meta-Analysis. Neuroepidemiology 46:292–300. https://doi.org/10.1159/000445751.

69. Whitmer RA, Gunderson EP, Barrett-Connor E, et al Obesity in middle age and future risk of dementia: a 27 year longitudinal population based study. BMJ 2005 Jun 11;330(7504):1360: https://doi.org/10.1136/bmj.38446.466238.E0.

70. Salas-Venegas V, Flores-Torres RP, Rodríguez-Cortés YM, et al (2022) The Obese Brain: Mechanisms of Systemic and Local Inflammation. and Interventions to Reverse the Cognitive Deficit Front Integr Neurosci 16:. https://doi.org/10.3389/fnint.2022.798995.

71. Espeland MA, Carmichael O, Hayden K, et al (2018) Action for Health In Diabetes Brain Magnetic Resonance Imaging (Look AHEAD Brain) and Action for Health Movement and Memory Ancillary Study Research Groups. Long-term Impact of Weight Loss Intervention on Changes in Cognitive Function: Exploratory Analyses from the Action for Health in Diabetes Randomized Controlled Clinical Trial. J Gerontol A Biol Sci Med Sci 14;73(4):484-491:. https://doi.org/10.1093/gerona/glx165.

72. Spitznagel MB, Hawkins M, Alosco M, et al (2015) Neurocognitive Effects of Obesity and Bariatric Surgery. Eur Eat Disord Rev. https://doi.org/10.1002/erv.2393.

73. Blasco BV, García-Jiménez J, Bodoano I, Gutiérrez-Rojas L (2020) Obesity and Depression: Its Prevalence and Influence as a Prognostic Factor: A Systematic Review. Psychiatry Investig Aug;17(8):715-724: https://doi.org/10.30773/pi.2020.0099.

74. Baune BT, Smith E, Reppermund S, et al (2012) Inflammatory biomarkers predict depressive, but not anxiety symptoms during aging: the prospective Sydney Memory and Aging Study. Psychoneuroendocrinology Sep;37(9):1521-30: https://doi.org/10.1016/j.psyneuen.2012.02.006.

75. Lv N, Kringle EA, Ma J (2022) Integrated Behavioral Interventions for Adults with Comorbid Obesity and Depression: a Systematic Review. Curr Diab Rep Apr;22(4):157-168: https://doi.org/10.1007/s11892-022-01458-z.

76. St Sauver JL, Boyd CM, Grossardt BR (2015) Risk of developing multimorbidity across all ages in an historical cohort study: differences by sex and ethnicity. BMJ Open 5:. https://doi.org/10.1136/bmjopen-2014-006413

77. Messier SP (2008) Obesity and osteoarthritis: disease genesis and nonpharmacologic weight management. Rheum Dis Clin North Am Aug;34(3):713-29: https://doi.org/10.1016/j.rdc.2008.04.007.

78. Goldring MB (2000) Osteoarthritis and cartilage: the role of cytokines. Curr Rheumatol Rep Dec;2(6):459-65: https://doi.org/10.1007/s11926-000-0021-y.

79. Robson EK, Hodder RK, Kamper SJ, et al (2020) Effectiveness of Weight-Loss Interventions for Reducing Pain and Disability in People With Common Musculoskeletal Disorders: A Systematic Review With Meta-Analysis. J Orthop Sports Phys Ther Jun;50(6):319-333: https://doi.org/10.2519/jospt.2020.9041.

80. Tang X, Liu G, Kang J, et al (2013) Obesity and risk of hip fracture in adults: a meta-analysis of prospective cohort studies. PLoS One 12;8(4):e55077: https://doi.org/10.1371/journal.pone.0055077.

81. Sadeghi O, Saneei P, Nasiri M, et al (2017) Abdominal Obesity and Risk of Hip Fracture: A Systematic Review and Meta-Analysis of Prospective Studies. Adv Nutr 15;8(5):728-738:. https://doi.org/10.3945/an.117.015545.

82. Cauley JA, Barbour KE, Harrison SL, et al (2016) Inflammatory Markers and the Risk of Hip and Vertebral Fractures in Men: the Osteoporotic Fractures in Men (MrOS. J Bone Miner Res Dec;31(12):2129-2138: https://doi.org/10.1002/jbmr.2905.

83. Barbour KE, Lui LY, Ensrud KE, et al (2014) Study of Osteoporotic Fractures (SOF) Research Group. Inflammatory markers and risk of hip fracture in older white women: the study of osteoporotic fractures. J Bone Miner Res Sep;29(9):2057-64: https://doi.org/10.1002/jbmr.2245.

84. Johnson KC, Bray GA, Cheskin LJ, et al (2017) The Effect of Intentional Weight Loss on Fracture Risk in Persons With Diabetes: Results From the Look AHEAD Randomized Clinical Trial. J Bone Miner Res. https://doi.org/10.1002/jbmr.3214.

85. Gale CR, Martyn CN, Cooper C, Sayer AA (2007) Grip strength, body composition, and mortality. Int J Epidemiol Feb;36(1):228-35: https://doi.org/10.1093/ije/dyl224.

86. Brinkley TE, Leng X, Miller ME, et al (2009) Chronic inflammation is associated with low physical function in older adults across multiple comorbidities. J Gerontol A Biol Sci Med Sci 64:455–461. https://doi.org/10.1093/gerona/gln038

87. Figgins E, Choi YH, Speechley M, Montero-Odasso M (2021) Associations Between Potentially Modifiable and Nonmodifiable Risk Factors and Gait Speed in Middle- and Older-Aged Adults: Results From the Canadian Longitudinal Study on Aging. J Gerontol A Biol Sci Med Sci 13;76(10):e253-e263:. https://doi.org/10.1093/gerona/glab008.

88. Houston DK, Neiberg RH, Miller ME, et al (2018) Physical Function Following a Long-Term Lifestyle Intervention Among Middle Aged and Older Adults With Type 2 Diabetes: The Look AHEAD Study. The Journals of Gerontology: Series A 73:1552–1559. https://doi.org/10.1093/gerona/glx204

89. García-Esquinas E, José García-García F, León-Muñoz LM, et al (2015) Obesity, fat distribution, and risk of frailty in two population-based cohorts of older adults in Spain. Obesity Apr;23(4):847-55: https://doi.org/10.1002/oby.21013.

90. Blaum CS, Xue QL, Michelon E, et al (2005) The Association Between Obesity and the Frailty Syndrome in Older Women: The Women’s Health and Aging Studies. J American Geriatrics Society 53:927–934. https://doi.org/10.1111/j.1532-5415.2005.53300.x

91. Nicklas BJ, Brinkley TE, Houston DK (2018) Effects of Caloric Restriction on Cardiorespiratory Fitness, Fatigue, and Disability Responses to Aerobic Exercise in Older Adults With Obesity: A Randomized Controlled Trial. J Gerontol Ser A. https://doi.org/10.1093/gerona/gly159

92. Silva JC, Moraes ZV, Silva C, et al (2014) Understanding red blood cell parameters in the context of the frailty phenotype: interpretations of the FIBRA (Frailty in Brazilian Seniors) study. Arch Gerontol Geriatr. https://doi.org/10.1016/j.archger.2014.07.014.

93. Nicklas BJ, Chmelo E, Delbono O, et al (2015) Effects of resistance training with and without caloric restriction on physical function and mobility in overweight and obese older adults: a randomized controlled trial. Am J Clin Nutr 101:991–999

94. Diago-Galmés A, Guillamon-Escudero C, Tenías-Burillo JM, et al (2023) Sarcopenic Obesity in Community-Dwelling Spanish Adults Older than 65 Years. Nutrients 27;15(23):4932: https://doi.org/10.3390/nu15234932.

95. Ryan DH, Espeland MA, Foster GD, et al (2003) Look AHEAD (Action for Health in Diabetes): design and methods for a clinical trial of weight loss for the prevention of cardiovascular disease in type 2 diabetes. Control Clin Trials 24:610–628

96. Kritchevsky SB, Lovato L, Handing EP, et al (2017) Exercise’s effect on mobility disability in older adults with and without obesity: The LIFE study randomized clinical trial. Obesity Jul;25(7):1199-1205: https://doi.org/10.1002/oby.21860.

97. Penninx BW, Kritchevsky SB, Newman AB, et al (2004) Inflammatory markers and incident mobility limitation in the elderly. J Am Geriatr Soc Jul;52(7):1105-13: https://doi.org/10.1111/j.1532-5415.2004.52308.x.

98. Rejeski WJ, Ip EH, Bertoni AG, et al (2012) Lifestyle change and mobility in obese adults with type 2 diabetes. N Engl J Med 29;366(13):1209–17:. https://doi.org/10.1056/NEJMoa1110294.
